# Promoting Physical Activity in a Spanish-Speaking Latina Population of Low Socioeconomic Status With Chronic Neurological Disorders: Proof-of-Concept Study

**DOI:** 10.2196/34312

**Published:** 2022-04-20

**Authors:** Alexander Garbin, Jesús Díaz, Vy Bui, Janina Morrison, Beth E Fisher, Carina Palacios, Ingrid Estrada-Darley, Danielle Haase, David Wing, Lilyana Amezcua, Michael W Jakowec, Charles Kaplan, Giselle Petzinger

**Affiliations:** 1 Division of Biokinesiology and Physical Therapy University of Southern California Los Angeles, CA United States; 2 Veterans Affairs Eastern Colorado Geriatric Research, Education, and Clinical Center Veterans Affairs Eastern Colorado Health Care System Aurora, CO United States; 3 Physical Therapy Program Department of Physical Medicine and Rehabilitation University of Colorado Anschutz Medical Campus Aurora, CO United States; 4 Chan Division of Occupational Science and Occupational Therapy University of Southern California Los Angeles, CA United States; 5 Suzanne Dworak-Peck School of Social Work University of Southern California Los Angeles, CA United States; 6 Primary Care Internal Medicine The Wellness Center Los Angeles County + University of Southern California Medical Center Los Angeles, CA United States; 7 Department of Neurology University of Southern California Los Angeles, CA United States; 8 Pardee RAND Graduate School Santa Monica, CA United States; 9 Exercise and Physical Activity Resource Center University of California at San Diego San Diego, CA United States

**Keywords:** exercise, quality of life, motivation, promotion, community study, clinical trial

## Abstract

**Background:**

Physical activity (PA) is known to improve quality of life (QoL) as well as reduce mortality and disease progression in individuals with chronic neurological disorders. However, Latina women are less likely to participate in recommended levels of PA due to common socioeconomic barriers, including limited resources and access to exercise programs. Therefore, we developed a community-based intervention with activity monitoring and behavioral coaching to target these barriers and facilitate sustained participation in an exercise program promoting PA.

**Objective:**

The aim of this study was to determine the feasibility and efficacy of a community-based intervention to promote PA through self-monitoring via a Fitbit and behavioral coaching among Latina participants with chronic neurological disorders.

**Methods:**

We conducted a proof-of-concept study among 21 Spanish-speaking Latina participants recruited from the Los Angeles County and University of Southern California (LAC+USC) neurology clinic; participants enrolled in the 16-week intervention at The Wellness Center at The Historic General Hospital in Los Angeles. Demographic data were assessed at baseline. Feasibility was defined by participant attrition and Fitbit adherence. PA promotion was determined by examining change in time spent performing moderate-to-vigorous PA (MVPA) over the 16-week period. The effect of behavioral coaching was assessed by quantifying the difference in MVPA on days when coaching occurred versus on days without coaching. Change in psychometric measures (baseline vs postintervention) and medical center visits (16 weeks preintervention vs during the intervention) were also examined.

**Results:**

Participants were of low socioeconomic status and acculturation. A total of 19 out of 21 (90%) participants completed the study (attrition 10%), with high Fitbit wear adherence (mean 90.31%, SD 10.12%). Time performing MVPA gradually increased by a mean of 0.16 (SD 0.23) minutes per day (*P*<.001), which was equivalent to an increase of approximately 18 minutes in MVPA over the course of the 16-week study period. Behavioral coaching enhanced intervention effectiveness as evidenced by a higher time spent on MVPA on days when coaching occurred via phone (37 min/day, *P*=.02) and in person (45.5 min/day, *P*=.01) relative to days without coaching (24 min/day). Participants improved their illness perception (effect size *g*=0.30) and self-rated QoL (effect size *g*=0.32). Additionally, a reduction in the number of medical center visits was observed (effect size *r*=0.44), and this reduction was associated with a positive change in step count during the study period (*P.*=04).

**Conclusions:**

Self-monitoring with behavioral coaching is a feasible community-based intervention for PA promotion among Latina women of low socioeconomic status with chronic neurological conditions. PA is known to be important for brain health in neurological conditions but remains relatively unexplored in minority populations.

**Trial Registration:**

ClinicalTrials.gov NCT04820153; https://clinicaltrials.gov/ct2/show/NCT04820153

## Introduction

In the last decade, studies have established the vital role of physical activity (PA), particularly sustained moderate-to-vigorous PA (MVPA), in chronic neurological disorders due to its ability to improve neurological outcomes, quality of life (QoL), and reduce disease progression [1-5]. Currently, the generalizability of these findings to US minority populations and different socioeconomic status groups in the United States is unclear given the fact that the vast majority of clinical research has been predominantly conducted on White males with affordable access to medical care.

Studies examining individuals who are regularly engaged in exercise in the United States have indicated that lack of PA is prevalent in Latinx groups compared to other ethnic groups [6]. In terms of sex, a high percentage (74%) of Latina women do not participate in exercise activities, and this number increases with age [7,8]. While there are numerous reasons for this occurrence, a substantial contributor includes the lack of resources and accessibility to programs that promote PA and exercise [9]. Therefore, it is paramount that clinical studies focus on development of accessible interventions that are capable of increasing PA in underrepresented communities and ethnic groups. One potential approach includes the implementation of a community-based intervention consisting of activity monitoring by personal activity monitors, such as wrist-worn Fitbit devices, with behavioral coaching. Numerous studies have demonstrated that this approach is impactful and successfully promotes PA among healthy individuals as well as those with medical conditions, including obesity, diabetes, and neurological disorders [10-12]. It is yet to be established whether specific motivational strategies can demonstrate efficacy in a Latina population among individuals with chronic neurological conditions.

The purpose of this proof-of-concept study was to examine the feasibility and efficacy of a community-based wellness center intervention to promote PA in a Latina population with chronic neurological disorders.

## Methods

### Ethical Considerations

The University of Southern California Institutional Review Board (IRB) approved all study procedures (IRB No. HS-18-00993). The funder did not require registration; however, the trial was retrospectively registered at ClinicalTrials.gov (NCT04820153) for publication. The authors confirm that any related clinical trials have been registered. All study participants provided written informed consent.

### Study Participants

The target sample size for this proof-of-concept study was 20 participants, as this is similar to sample sizes used in previous preliminary Fitbit wear studies [11,13]. Inclusion criteria included the following: (1) diagnosed with a neurological disorder, (2) able to use the Fitbit activity monitor as determined by participant self-report after receiving instructions from a study coordinator, (3) own a smartphone device, (4) ambulatory without assistance, (5) able to provide informed consent, (6) live within commuting distance to The Wellness Center (TWC) at The Historic General Hospital in Los Angeles, (7) Spanish speaking, and (8) have internet access. Exclusion criteria included any physical condition that precluded engagement in exercise or PA.

### Study Design

#### Recruitment and Community Site

**Figure 1:**
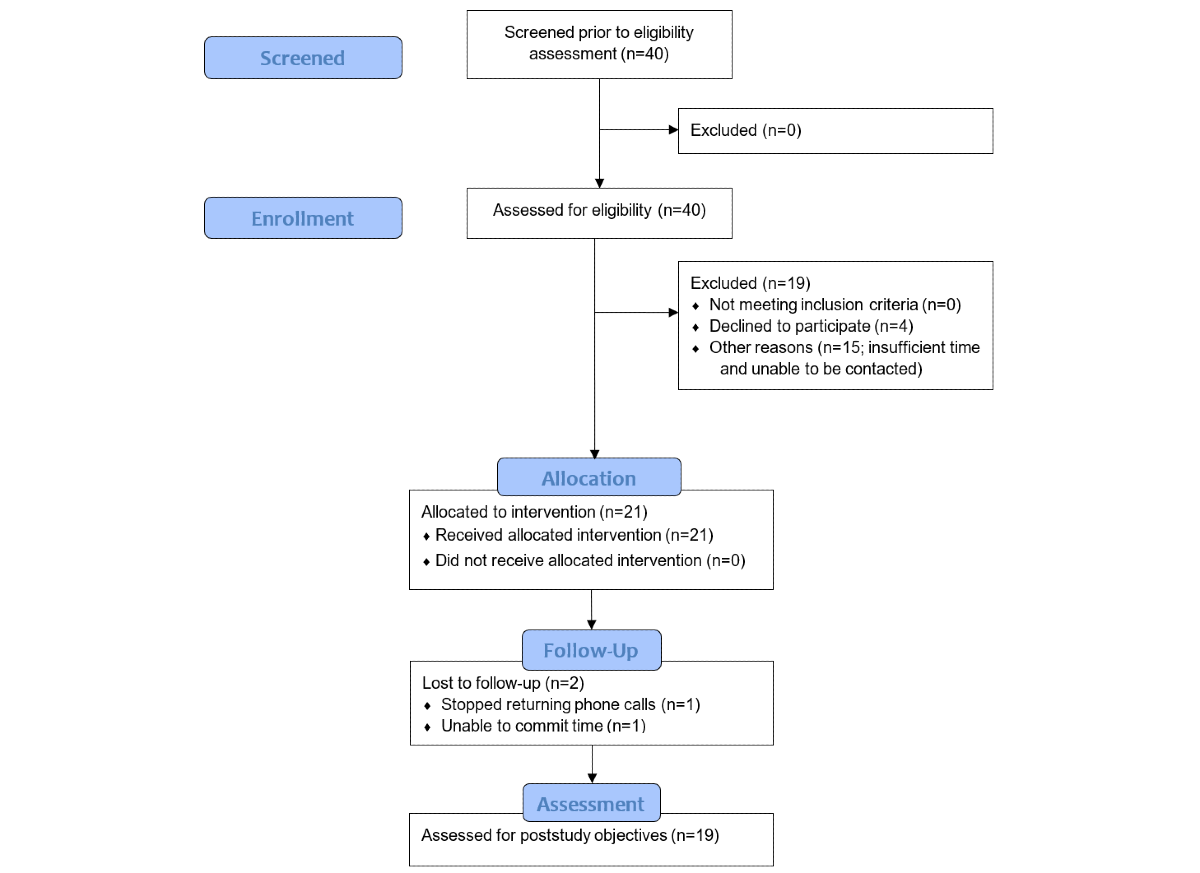
CONSORT (Consolidated Standards of Reporting Trials) flow diagram.

Candidate Latina participants living in Central and East Los Angeles were identified by bilingual attending neurologists in the Department of Neurology; candidates were identified in the clinic at the Los Angeles County and University of Southern California (LAC+USC) Medical Center. The LAC+USC neurology clinic primarily serves a Latinx population of low socioeconomic status who have a wide spectrum of neurological conditions, including epilepsy, chronic headache, migraine, stroke, Parkinson disease, and multiple sclerosis. A total of 21 individuals consented to participate in the study (Figure 1); these participants enrolled in the study at TWC, a community center located within the Boyle Heights community of East Los Angeles that offers free health and wellness classes and programs. TWC was founded in 2014 and provides a wide range of services to patients, caregivers, and their families.

#### Community-Based PA Intervention

The study consisted of a 16-week community-based intervention aimed at promoting PA through behavioral coaching and self-monitoring via a Fitbit Alta HR activity device (Fitbit, Inc). Intervention length was based on the success of similar PA promotion studies using Fitbit devices among healthy individuals and those with various medical disorders [11,12]. Upon enrollment, individuals received Fitbit personal activity monitors and were instructed on their usage and maintenance. Participants were notified at the commencement of the study that they would be allowed to keep their Fitbit activity monitor upon study completion.

#### Fitbit Personal Activity Monitors

Individuals were provided with the Fitbit device and charger and were asked to wear the Fitbit device every day during the 16-week study except when bathing or swimming. Individuals had the Fitbit app program set up on their smartphones, thus allowing for continuous self-monitoring of PA by the Fitbit device and longitudinal monitoring to collect data by the Fitbit app throughout the 16-week study period. Participants’ Fitbit wear compliance was encouraged by a once-a-week behavioral coaching phone call. Monitored PA data from the Fitbit were downloaded weekly.

#### Behavioral Coaching

Navigators at TWC are Latina women who come from Central and East Los Angeles communities. Their role at the TWC is to perform outreach, perform health coaching, and guide members to health resources. Within the study, navigators provided assistance by motivating participants and providing support for any technological issues. Behavioral coaching consisted of the following: (1) 1 hour of Lifestyle Redesign at baseline and at week 4 with an occupational therapist of Latino background located at TWC and (2) weekly contacts of 5 to 10 minutes in length from TWC navigators by telephone. Lifestyle Redesign is a well-established program for promoting behavioral modification including PA by addressing physical, psychosocial, and environmental barriers to health. It incorporates self-care strategies and goal setting and has been validated in Latinx populations [14]. For this intervention, goal setting centered on supporting increased PA for each participant. This was accomplished by asking participants at baseline what activities they wanted to do to increase PA in their lives and by subsequently collaborating on plans to incorporate these activities into their daily lives. The weekly contacts from navigators occurred either in person or via phone and focused on promotion of PA, Fitbit wear, and goal review.

### Study Participant Characteristics

#### Sociodemographics

Sociodemographic information included age, sex, primary language, highest education level achieved, health perception, neurological diagnoses, primary care provider status, insurance status, housing status, and food insecurity. Zip code data were collected and subsequently assessed to infer income category [15]. Specifically, median incomes for participant zip codes were compared to the Los Angeles County (LAC) median income and were categorized as very low income (<50% of LAC median income), low income (50%-80% of LAC median income), or not low income (>80% of LAC median income) per the US Department of Housing and Urban Development recommendations.

#### Acculturation

Acculturation is defined as the cultural modification of an individual or group by adapting to or borrowing traits from one culture and displacing characteristics, habits, attitudes, and mode of life of another culture. Acculturation was assessed through the Short Acculturation Scale for Hispanics (SASH), a 12-item questionnaire where questions are answered using a 5-point Likert scale. The SASH is used to assess the level to which participants acculturate or adopt the attitudes, values, customs, beliefs, and behaviors of another culture. The scale provides an overall score as well as three subscale scores in language use, media preference, and ethnic and social relations. A higher score indicates higher acculturation with the culture of the United States [16]. 

### Outcomes Measures

#### Fitbit Data Measures

Objective PA and wear-time data gathered from the Fitbit device were stored, visualized, and aggregated through a commercially available service (Fitabase Inc, Small Steps Labs) using Health Insurance Portability and Accountability Act–compliant deidentified unique participant emails. PA measures included step count and time spent on (1) sedentary activity; (2) light, moderate, and vigorous PA (LMVPA); or (3) MVPA. Activity intensity was quantified via a Fitbit proprietary algorithm based on metabolic equivalents of task. To evaluate compliance to Fitbit device wearing, day wear was calculated as the “total wear minutes” (derived from minutes where Fitbit captured heart rate value) minus the “in-bed minutes” (derived from the Fitbit proprietary sleep detection algorithm). Data for each day were first inspected for technical adherence, defined as having accurate sleep data (ie, in-bed minutes greater than 180 minutes if day wear minutes were greater than 1080 minutes out of the maximum of 1440 minutes per day) and activity data (ie, LMVPA minutes greater than 0 minutes if day wear minutes were greater than 600 minutes [10 hours]). Personal adherence, defined as greater than 10 hours of Fitbit wear per day, was subsequently assessed for days that were technically adherent. Fitbit-recorded PA measures were only analyzed for days with valid technical adherence and personal adherence.

#### Quality of Life Scale

The Quality of Life Scale (QoLS) was used to assess an individual’s view of their health, how well they feel, and how well they were able to complete their usual activities [17]. The QoLS is a 16-item assessment, with items rated on a 7-point Likert scale, measuring five life domains: (1) material and physical well-being; (2) relationships with others; (3) social, community, and civic activities; (4) personal development and fulfillment; and (5) recreation. A higher score is indicative of a higher perceived QoL.

#### Illness Perception Scale

The Brief Illness Perception Questionnaire (BIPQ) was administered to assess individuals’ perceptions of their health and well-being [18]. The BIPQ is a 9-item questionnaire, with questions rated on a 10-point Likert scale. Each question assesses one dimension of illness perception, including cognitive and emotional representation, level of personal and treatment control, and sense of identity and concern. A higher score indicates a more threatening view of a participant’s illness.

#### System Usability Scale

The System Usability Scale was used to ascertain an individual’s opinion on the usability of the Fitbit phone app, Fitbit activity monitor, and the combination of the Fitbit device and app [19].

#### LAC+USC Medical Center Visits

The total number of outpatient and inpatient visits to the LAC+USC Medical Center was determined during the 16-week period prior to study initiation and during the period of the study intervention using electronic medical records. LAC+USC Medical Center visits were monitored to gather insight into participants’ overall health behaviors as well as to specifically understand the impact of PA promotion on health care use.

### Data Analysis

Analyses were completed using the statistical analysis package R (version 3.6.0; The R Foundation) [20]. The mean and SD of the data were calculated to describe the sociodemographic characteristics, scale outcomes, and Fitbit-recorded PA. Individuals’ rates of change in Fitbit-recorded PA measures were quantified by an activity (eg, MVPA) by day regression slope. Promotion of PA was determined by examining whether time spent performing MVPA increased throughout the 16-week intervention period or stayed stationary. To accomplish this, a Kwiatkowski-Phillips-Schmidt-Shin (KPSS) test was performed on the time-series MVPA data. If the KPSS test was significant, indicating that MVPA data were nonstationary, an MVPA by time (day) Pearson correlation was calculated to determine whether time spent performing MVPA significantly increased or decreased throughout the intervention period. A Kruskal-Wallis test was employed to evaluate differences in PA measures on days with varying behavioral coaching contact types (ie, phone, in person, or no contact). When appropriate, the Dunn test was performed to determine the locus of significance [21]. Changes in scale outcomes from baseline to postintervention and medical center visits from baseline to the study period were assessed by paired *t* tests (2-tailed) or Wilcoxon signed-rank tests depending on normality. Hedges *g* was used to calculate effect size when changes were quantified by paired *t* tests. The *r* statistic—obtained from the formula *r* = *Z* statistic / sqrt(n)—was used with the Wilcoxon signed-rank test. A Pearson correlation assessed the relationships between participant-level rates of change in PA measures and changes in medical center visits. An α level of less than .05 was considered statistically significant.

## Results

### Participant Characteristics

Key demographics of study individuals are presented in Table 1. A total of 21 Latina participants were enrolled, and 19 (90%) of them completed the study, giving an attrition rate of 10%. The mean age of the sample was 47 years (SD 9.0, range 29-64). Neurological diagnoses included chronic headaches and migraines (n=13, 68%), Parkinson disease (n=2, 11%), multiple sclerosis (n=2, 11%), and trigeminal neuralgia (n=2, 11%). The majority of individuals were considered to be of low socioeconomic status. A total of 76% (13/17) of participants were living in low- or very low–income areas; 63% (12/19) completed less than a high school–level education; 41% (7/17) reported housing insecurities, defined as not having housing or being worried about losing housing; and 53% (9/17) reported food insecurities, defined as being worried about running out of food before having money to buy more. Overall, study participants had low acculturation to United States culture, as evidenced by a mean acculturation score of 1.81 (SD 0.65).

**Table 1:** Sociodemographic characteristics of the study participants.

Characteristics								Values (N=19)
Age (years), mean (SD, range)								47 (9.0, 29-64)
Sex (female), n (%)								19 (100)
**Neurological diagnosis, n (%)**								
	Chronic headaches and migraines							13 (68)
	Parkinson disease							2 (11)
	Multiple sclerosis							2 (11)
	Trigeminal neuralgia							2 (11)
**Primary language, n (%)**								
	Spanish							18 (95)
	English							1 (5)
**Self-reported overall health (n=17), n (%)**								
	Excellent or good							5 (29)
	Fair or poor							12 (71)
**Socioeconomic status (n=17), n (%)**								
	**Zip code family income category**							
			Not low income				4 (24)	
			Low income				10 (59)	
			Very low income				3 (18)	
	**Highest education level achieved**							
					≤11th grade			12 (63)
					≥12th grade			7 (37)
	**Housing status (n=17), n (%)**							
		Have housing						10 (59)
		Worried about losing housing or do not have housing						7 (41)
	**Insurance status**							
						Public insurance	11 (58)	
						Other, none, or do not know	8 (42)	
	**Primary care provider**							
				Yes				13 (68)
				No				6 (32)
	**Food insecurity (n=17), n (%)**							
		Yes						9 (53)
		No						8 (47)
**Acculturation, as measured by the SASH^a^, mean (SD)**								
	Average score							1.81 (0.56)
	Language use score							1.52 (0.73)
	Media preference score							2.09 (1.10)
	Ethnic and social relations score							1.96 (0.53)

^a^SASH: Short Acculturation Scale for Hispanics. Total average and subscale scores range from 1 to 5. Higher scores indicate higher acculturation.

### Intervention Adherence and Safety

A total of 19 out of 21 (90%) participants completed the intervention study, while 2 (10%) participants were lost to follow-up. No adverse events were reported by participants. A mean of 93.6% (SD 9.5%) of days during the intervention period had valid technical adherence. There was also high personal adherence, as the average participant wore their Fitbit for 10 hours or greater on a mean of 90.31% (SD 10.12%) of days throughout the 16-week trial (mean 5.95, SD 1.02 days/week). Further, while the recommended day wear time was 10 hours, the average participant wore their Fitbit for a mean of 15.44 (SD 1.06) day waking hours. Participants rated the usability of the Fitbit device and app as good (score 71.4) to excellent (score 85.5) [22], potentially reflecting the high adherence (Table 2).

**Table 2:** Fitbit wear adherence, physical activity, and system usability feedback.

Measure					Mean (SD)
**Adherence**					
			Technical adherence (% days)	93.6 (9.50)	
			Personal adherence (% days)	90.31 (10.12)	
			Valid personal adherence days per week	5.95 (1.02)	
			Wear minutes per day waking hour	926.61 (63.27)	
**Physical activity per day**					
		Number of steps		9859.99 (2616.22)	
		Sedentary minutes		583.51 (80.15)	
		LMVPA^a^ minutes		336.84 (80.09)	
		MVPA^b^ minutes		36.63 (18.26)	
**Individual rate of change per day**					
	Number of steps			–0.91 (27.69)	
	Sedentary minutes			–0.07 (0.62)	
	LMVPA minutes			0 (0.71)	
	MVPA minutes			0.16 (0.23)	
**System Usability Scale^c^ feedback (%)**					
	Fitbit device			76.3 (7.4)	
	Fitbit app			78.4 (8.0)	
	Fitbit device and app combined			82.9 (10.5)	

^a^LMVPA: light, moderate, and vigorous physical activity.

^b^MVPA: moderate-to-vigorous physical activity.

^c^Higher percentages of feedback for the System Usability Scale indicate higher usability.

### Promotion of Physical Activity 

**Figure 2:**
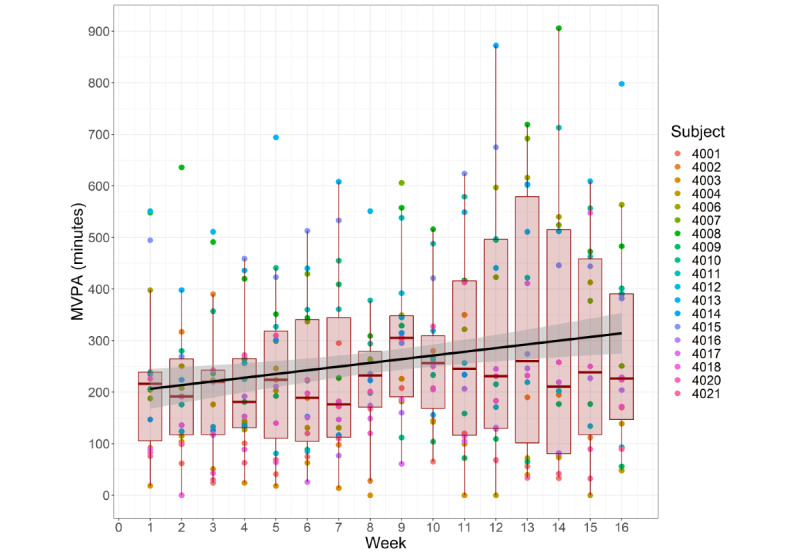
Moderate-to-vigorous physical activity (MVPA) throughout the 16-week intervention. Each dot represents a single week, with the color of the dot specifying the participant. Numbers beside the colored dots represent participant numbers. The box plots detail weekly medians (horizontal red lines) and quartile minutes performing MVPA for the group, while the black line illustrates the slope of MVPA over time for the group.

The average participant increased their time spent performing MVPA by a mean of 0.16 (SD 0.23) minutes per day over the 16-week study period, which was equivalent to a cumulative increase of approximately 18 minutes. PA was promoted as indicated by a significant KPSS test (*P*=.01 and subsequent significant positive Pearson correlation [*r*=0.457, 95% CI 0.297-0.592]; *P*<.001) detailing that time spent performing MVPA increased throughout the 16-week study period (Figure 2). No changes were observed during the intervention period for other PA measures (Table 2).

### Impact of Behavioral Coaching

**Figure 3:**
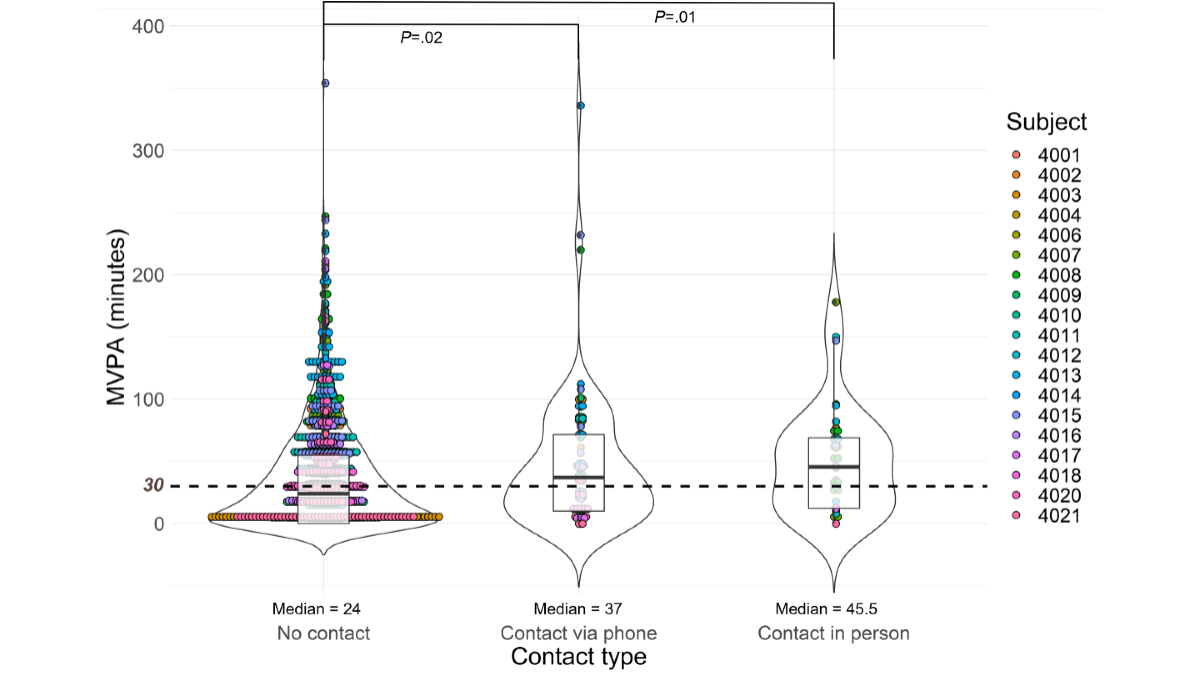
The effect of contact type on daily moderate-to-vigorous physical activity (MVPA) minutes. Each dot represents a single day, with the color of the dot specifying the participant. Numbers beside the colored dots represent participant numbers. The violin outline details the overall distribution of each contact type condition, while the box plots depict the medians and quartiles. The horizontal dashed line at 30 MVPA minutes illustrates the recommended number of MVPA minutes per day.

Participants were contacted for behavioral coaching an average of 5.26 (SD 1.88) times by phone and 2.11 (SD 1.63) times in person over the 16-week study period. A Kruskal-Wallis test revealed that median steps and MVPA were greater on days when individuals were contacted via phone (10,495 steps/day, IQR 8547.5-12,979.5, *P=.*03; MVPA: 37 min/day, IQR 10-71.5, *P=.*02) and in person (11,369.5 steps/day, IQR 9886.23-14,214.8, *P=.*01; MVPA: 45.5 min/day, IQR 12.3-69, *P=.*01) compared to days when individuals were not contacted (10,161 steps/day, IQR 6639-12,371; MVPA: 24 min/day, IQR 0-55; Figure 3). Median sedentary minutes were reduced on days when participants were contacted by phone (561 min/day, IQR 470-639.5, *P*=.04) but not in person (580 min/day, IQR 516.3-637.8, *P*=.43) relative to days with no contact (580.5 min/day, IQR 493-669). No differences were seen in LMVPA regardless of contact (contact by phone: 351 min/day, IQR 282-416, *P=.*10; contact in person: 364.5 min/day, IQR 278.5-422.8, *P=.*16; no contact: 334 min/day, IQR 255-408).

### Scales

**Figure 4:**
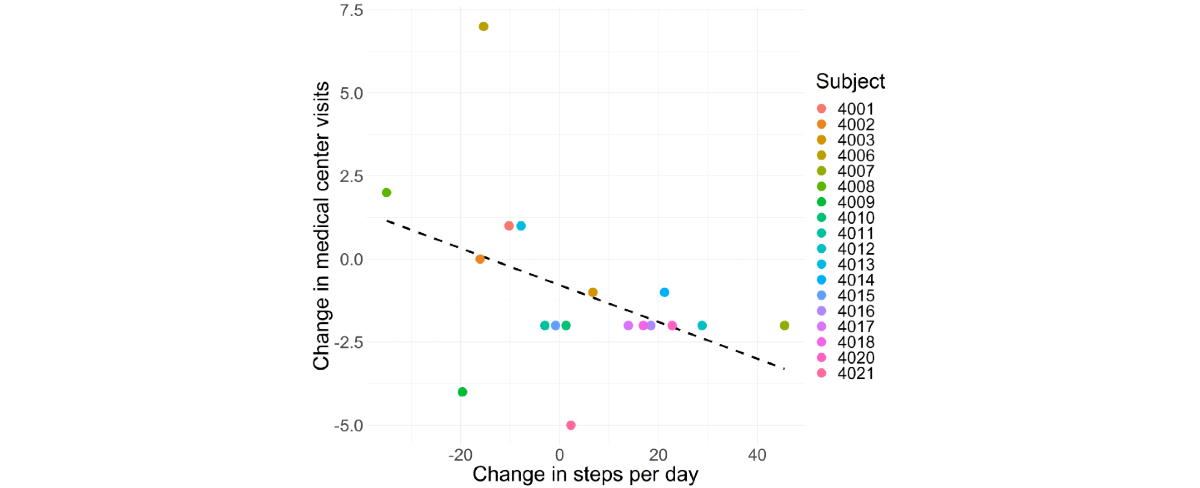
The association between change in medical center visits with rate of change in steps. A negative change in medical center visits indicates that a participant went to the medical center fewer times during the study period than during the period 16 weeks prior to study initiation. Numbers beside the colored dots represent participant numbers.

Small effect sizes were observed for improvement in BIPQ scores (*g*=0.30, *P=.*19) and increases in QoLS scores (*g*=0.32, *P=.*16). A medium effect size was observed for reductions in visits to the LAC+USC Medical Center (effect size *r*=0.44, *P=.*06; Table 3). A positive rate of change in steps correlated with reduced LAC+USC Medical Center visits (correlation coefficient *r*=–0.47, *P=.*04; Figure 4). There was no correlation between change in medical center visits and change in other PA measures.

**Table 3:** Measurements before, during, and after the intervention study period.

Measure	First measurement^a^, mean (SD)	Second measurement^b^, mean (SD)	*P* value	Effect size
Brief Illness Perception Questionnaire score^c^	43.05 (13.11)	39.21 (14.32)	.19	0.30^d^
Quality of Life Scale score^e^	82.37 (19.05)	87.53 (11.72)	.16	0.32^d^
Number of medical center visits (4 months)	4.32 (2.43)	3.47 (2.84)	.06	0.44^f^

^a^The first measurements for the scales took place at baseline, whereas those for medical center visits took place during a period 16 weeks prior to the start of the study.

^b^The second measurements for the scales took place after the intervention study period, whereas those for medical center visits took place during the 16-week study.

^c^Total scores range from 0 to 80; a higher score indicates a more threatening view of a participant’s illness.

^d^This effect size was Hedges *g*.

^e^Total scores range from 16 to 112; a higher score indicates a higher perceived quality of life.

^f^This effect size was the *r* statistic.

## Discussion

### Principal Findings

The aim of this proof-of-concept study was to assess the feasibility and efficacy of a community-based intervention to promote PA through (1) a wearable Fitbit and (2) behavioral coaching in a sample of Latina women of low socioeconomic status with chronic neurological disorders recruited from the LAC+USC county outpatient neurology clinic. Studies have demonstrated the importance of PA and exercise in reducing morbidity and improving QoL for individuals with chronic neurological disorders, including Parkinson disease, multiple sclerosis, and chronic pain [1,2,4,23]. However, these studies typically do not include individuals from minority populations of low socioeconomic status. In general, underserved populations, particularly Latina women, are also less physically active due to numerous barriers to exercise, placing them at high risk for disease burdens associated with chronic illness [6-8]. These barriers include environmental factors, such as great distances with limited public transportation; low socioeconomic status; long working hours; lack of health literacy; limited social support, including extensive family and caregiver demands; and lack of facilities, including gym spaces, outdoor parks, or recreational sites [9]. Health care–related resources delivered at the community level may remove these barriers by maximizing accessibility and redesigning lifestyles [24]. For example, behavioral coaching and encouragement delivered within the community can help individuals identify goals and develop strategies to incorporate PA into their daily lives that otherwise would have not been considered [14]. Additionally, recent personal activity monitoring technology, such as Fitbit devices, enable individuals to monitor their goals through continuous and goal-directed real-time feedback emphasizing self-selected PA rather than structured exercise.

This proof-of-concept study provided preliminary evidence that programs designed to promote PA through self-monitoring with a personal activity monitor, such as a Fitbit wearable device, in conjunction with behavioral coaching are feasible and impactful among Latina women of low socioeconomic status and acculturation. Specifically, we observed high adherence to Fitbit wear, consistent with similar intervention studies using the same personal adherence definition (10 waking hours/day) in samples largely consisting of non-Latina, White females [11]. Potential motivating factors underlying high adherence include (1) initial goal setting, (2) frequent contact with wellness center navigators [25] to motivate the participants and help with any technological issues, and (3) the incentive for individuals to keep the Fitbit after study completion. Further, it is likely that the community-based nature of the intervention facilitated adherence by reducing barriers to exercise commonly experienced by this population of low socioeconomic status.

Importantly, this study demonstrated the efficacy of the intervention to promote PA over a 16-week period. In general, participants experienced an increase of approximately 18 minutes in daily MVPA over the course of the 16-week period, which nears the World Health Organization recommendations for approximately 20 minutes of PA per day (150 minutes of PA/week) to improve general and brain health [26]. This increase in MVPA was steady (0.16 minutes/day), which suggests that these gains in PA are gradual in nature. Taken together, these findings suggest that reaching recommended MVPA levels may be an achievable and realistic goal. While an increase in MVPA has been observed in similar self-monitoring intervention studies [12,27], it is notable that Latina women and individuals with chronic neurological conditions typically do not achieve recommended levels of PA [7,28] and report many barriers to exercise [9,29]. 

Interestingly, this study demonstrates that the observed PA promotion was significantly influenced by behavioral coaching within the setting of a community wellness center. Specifically, both behavioral coaching by phone from a wellness center navigator and by in-person visits with an occupational therapist increased individuals’ activity as reflected by time spent performing MVPA and daily step count. This finding is consistent with previous reports demonstrating that community-based interventions can promote PA among Latina women, particularly when led by those within their own community [30,31]. While the use of Fitbit wearable devices as an intervention is rising in popularity and studies have demonstrated its benefit in promoting PA in the general population of women [11-13], our results support the importance of a community-based intervention model that combines Fitbit wear and behavioral coaching to promote PA for Latina women with low socioeconomic status and acculturation characteristics.

Group-level changes in the number of daily steps, illness perception, QoL, and clinical visits were not significantly altered in this study. However, further analyses revealed that an increase in daily step count during the study period was correlated with a reduction in clinical visits. Two potential drivers of this relationship may include either the following: (1) increasing step count to improve the health of individuals, thus reducing medical center visits, or (2) the degree of health-related problems that required medical center visits resulted in reduced step counts. While the former demonstrates the importance of interventions that increase step count, both potential drivers show how monitoring change in daily number of steps may inform health status and the need for clinical visits. In fact, previous work has found that increased step count during inpatient recovery from cancer surgery is predictive of reduced likelihood for hospital readmission [32]. Results from this preliminary study suggest that the predictive strength of daily step count extends to community-based activities. A larger-scale study aimed at examining the relationship between steps and medical center visits is required to better understand the precise underlying mechanisms of this association.

There are limitations to this study due to its nature as a proof-of-concept study assessing feasibility and efficacy of an intervention in a targeted population. The sample size was relatively small, drawn from a specific geographical area, and consisted of a narrow range of neurological disorders, thus limiting our ability to generalize our findings to a larger population of Latina women with chronic neurological disorders. However, this study can be used as a guide for a future large randomized clinical trial examining PA promotion in this population with specific neurological disorders, including individuals suffering from chronic pain, Parkinson disease, or multiple sclerosis. While not a limitation, variability, including a potential ceiling effect in some individuals, was observed in activity levels between participants throughout the 16-week study. This variability is unsurprising given the proof-of-concept nature of this study focusing on an understudied and underresourced population commonly at risk for adverse health conditions in which little is known about PA engagement. As such, the authors did not use exclusion criteria or stratify based on preintervention activity levels. An additional limitation includes a lack of prestudy PA measurements. Due to this limitation, it is not possible to determine whether the high initial activity levels and step counts observed in the study were due to an initial increase that was secondary to Fitbit wear or whether individuals were already physically active at a high level. Similarly, PA levels or other outcomes following cessation of the formal intervention period were not measured, so we cannot determine whether the long-term benefits of the intervention persisted. Lastly, participants did not keep sleep or activity logs, which resulted in an overreliance on Fitbit data to accurately record these metrics.

### Conclusions

This proof-of-concept study supports the feasibility and efficacy of a community-based intervention consisting of self-monitoring by a personal activity monitor, such as a Fitbit, combined with behavioral coaching by phone or in person promoting PA in a sample of Latina women with chronic neurological conditions. Behavioral coaching appears to enhance PA promotion when delivered by a culturally competent intervention team consisting of wellness center navigators from the community as well as an occupational therapist of Latino background. These initial results support the development of future large-scale intervention studies with the goal of providing evidence for the feasibility and effectiveness of PA promotion in this underrepresented population.

## Abbreviations

BIPQ: Brief Illness Perception Questionnaire
IRB: Institutional Review Board
KPSS: Kwiatkowski-Phillips-Schmidt-Shin
LAC: Los Angeles County
LAC+USC: Los Angeles County and University of Southern California
LMVPA: light, moderate, and vigorous physical activity
MVPA: moderate-to-vigorous physical activity
PA: physical activity
QoL: quality of life
QoLS: Quality of Life Scale
SASH: Short Acculturation Scale for Hispanics
TWC: The Wellness Center

## Data Availability

Access to data can be obtained by reasonable request to the contributing or senior authors (AG and GP).
